# Role of chest radiograph in MERS-Cov pneumonia: a single tertiary referral center experience in the United Arab Emirates

**DOI:** 10.1186/s43055-021-00517-x

**Published:** 2021-05-25

**Authors:** Karuna M. Das, Jamal Aldeen Alkoteesh, Mohamud Sheek-Hussein, Samira Ali Alzadjali, Mariam Tareq Alafeefi, Rajvir Singh, Yauhen Statsenko, Elpidoforos S. Soteriades, Vishwajeet Singh, Klaus Van Gorkom

**Affiliations:** 1 Department of Radiology, College of Medicine and Health Sciences, United Arab Emirates University, P.O. Box 17666, Al Ain, United Arab Emirates; 2 Department of Radiology, Al Ain Hospital, Al Ain, United Arab Emirates; 3 Institute of Public Health, College of Medicine and Health Sciences, United Arab Emirates University, Al Ain, United Arab Emirates; 4 Department of Biostatistics, All India Institute of Medical Sciences, New Delhi, India; 5 Department of Environmental Health, Environmental and Occupational Medicine and Epidemiology (EOME), Harvard T.H. Chan School of Public Health, Boston, MA USA

**Keywords:** MERS-Cov, Chest radiograph, ARDS

## Abstract

**Background:**

The Middle East respiratory syndrome coronavirus (MERS-Cov) continues to be a source of concern due to intermittent outbreaks. Serial chest radiographic changes in MERS-Cov patients were analyzed for various variables that could be compared to the patients’ final outcomes in a cluster of MERS-Cov patients and to identify a predictor of mortality in the United Arab Emirates.

**Results:**

A total of 44 MERS-Cov cases were reviewed. The mean age of the patients was 43.7 ± 14.7 years. The chest radiograph was abnormal in 14/44 (31.8%). The commonest radiology features include ground-glass opacities (seven of 14, 50%), ground-glass and consolidation (seven of 14, 50%), pleural effusion (eight of 14, 57.1%), and air bronchogram (three of 14, 21.4%). The mortality rate was 13.6% (six of 44); the deceased group (6 of 44, 13.6%) was associated with significantly higher incidence of mechanical ventilation (*p* < 0.001), pleural effusion (*p* < 0.001), chest radiographic score (8.90 ± 6.31, *p* < 0.001), and type 4 radiographic progression of disease (*p* < 0.001). A chest radiographic score at presentation was seen to be an independent and strong predictor of mortality (OR [95% confidence interval] 3.20 [1.35, 7.61]). The Cohen κ coefficient for the interobserver agreement was k = 0.89 (*p* = 0.001).

**Conclusion:**

The chest radiographic score, associated with a higher degree of disease progression (type 4), particularly in patients with old age or with comorbidity, may indicate a poorer prognosis in MERS-Cov infection, necessitating intensive care unit management or predicting impending death.

## Background

The Middle East respiratory syndrome coronavirus (MERS-Cov) was first described as a zoonotic disease in Saudi Arabia in 2012 and subsequently spread to countries in the Middle East and beyond the Arabian Peninsula [1]. Since 2012, UAE has reported 88 cases (including the patient-reported above) of MERS-Cov infection and 12 associated deaths [1]. From 2012 to July 2020, the total number of laboratory-confirmed MERS-Cov-infected cases reported globally by the WHO was 2562, with 881 associated deaths [1–3]. Approximately 55% of patients with MERS-Cov require admission to the intensive care unit due to respiratory failure, including the development of adult respiratory distress syndrome (ARDS) [4, 5]. ARDS frequently leads to a clinically significant reduction in health-related quality of life and is also associated with various radiological changes [6]. A recent study described chest radiograph abnormalities associated with MERS-Cov and demonstrated that sequential chest radiographic evaluation is a valuable tool for the early diagnosis and monitoring of disease progression [4].

The purpose of this study was to determine the role of various variables such as mechanical ventilation days, chest radiographic score, disease progression on radiographic imaging, and pleural effusion in predicting mortality in clusters of MERS-Cov patients in the United Arab Emirates.

## Methods

The study was conducted on 44 patients with positive MERS-Cov retrospectively from electronic medical records. The patient diagnosis was performed using RT-PCR and was based on the World Health Organization criteria [7]. All these 44 patients were recruited from the United Arab Emirates. The available chest radiographs were obtained between July 1, 2013, and September 2019.

The Institutional Review Board approved the study protocol, and the requirement for informed consent was waived due to the study’s retrospective nature. The inclusion criteria were as follows: admission to the hospital with symptoms suggestive of MERS-Cov without a history of other lung infectious diseases and has laboratory-confirmed MERS-Cov infection as determined by RT-PCR in any specimen or saliva. The exclusion criteria were the group of patients with flu-like symptoms with negative RT-PCR test for MERS-Cov and those patients without any initial chest radiographs. The study group of 44 patients was divided into two subgroups on the basis of the final outcome of recovery or death.

### Study protocol and data analysis

Digital radiography equipment (Mobilett Plus; Siemens, Erlangen, Germany) was used to obtain portable anteroposterior (AP) projection radiographs as per the local protocol. Two chest radiographs were routinely taken per day for very sick patients in the ICU and once every other day during the recovery stage. Two radiologists with more than 20 years of experience (KMD, JK) reviewed the chest radiographs independently. Any of the discrepant reports was assessed independently by an arbitrator (KG). All patients were evaluated based on the risk factors like mechanical ventilation days, chest radiographic score, pleural effusion, and chest radiographic disease progression pattern [4, 8]. The lung parenchyma and airways were evaluated for consolidation, ground-glass opacity (GGO), pleural effusion, cavitation, and pneumothorax [4, 8]. For the purpose of calculating the lung score, each lung was divided into three zones, and each zone was evaluated for disease process involvement. The development of MERS-Cov lesions within each lung zone was assigned a score ranging from 0 (normal) to 4 (complete involvement of one zone); a score of 24 indicated complete involvement of all six zones. The scores for each of the six zones in each chest radiographic study were added together to generate a cumulative chest radiographic score ranging from 0 to 24 based on the degree of lung parenchymal involvement. At the time of initial presentation and at the peak of disease activity, scores were taken. The disease progression pattern (types 1–4) was identified from chest radiographs described by Wong et al. [9]. Radiographic deterioration followed by complete improvement was used to define type 1 disease progression. Type 2 disease progression was defined as stable radiographic changes without discernible radiographic peaks or a decrease in overall mean lung involvement of less than 25%. The progression of type 3 disease was defined as fluctuating radiographic changes with at least two radiographic peaks separated by a period of mild remission, with remission defined as a level of mean lung parenchyma involvement that was more than 25% different from the peak level. Progressive radiographic deterioration was defined as type 4 progression. Several of the above parameters, including chest radiographic disease progression pattern (types 1–4), were compared between those who recovered and those who did not.

### Statistical analyses

The categorical variables were described using absolute or relative frequency distribution and quantitative variables using central tendency or location measures, such as mean (standard deviation) or median (interquartile range). The association between qualitative independent variables was assessed using the chi-square test or Fisher’s exact test as appropriate. To compare quantitative variables between the two groups (deceased and recovered), a *t*-test or Wilcoxon rank-sum test was used as appropriate. A logistic regression procedure was used to determine factors associated with mortality; the results are presented in the form of the odds ratio and corresponding 95% confidence interval and *p* value. The discrimination performance of the logistic regression was evaluated using the area under the curve (AUC). A *p-*value <0.05 (two tailed) was considered to be statistically significant. Statistical software, STATA/SE version 14.2 (Stata Corp LP, College Station, TX, USA), was used for the analysis. Interobserver reliability is calculated using Cohen’s kappa statistics (k) to see the degree of agreement between the two reviewers.

## Results

The present cohort includes 44 MERS-Cov patients with a mean age of 43.7 ± 14.7 years, range, 11–71 years, with M:F 28 males and 16 females. The demographic and clinical characteristics of MERS-Cov cases by mortality status are shown in Table 1. Forty-four patients of MERS-Cov were divided into two groups, those who survived (n = 38) and those who died (n = 6). Patients’ age in the deceased group was significantly higher than that seen in the recovered group (59.8 ± 14.2 vs. 41.16 ± 13.36 years, *p* = 0.008). The most common symptoms at the time of the presentation were fever (27%), cough (27%), dyspnea (9%), rhinitis (9%), and myalgia (4.5%). Associated comorbidity was noted in 24 of 44 (54.5%) of cases with hypertension (15 of 44, 34%), diabetes (ten of 44, 22.7%), heart disease (four of 44, 9%), obesity (three of 44, 7%), end-stage renal disease (three of 44, 7%), smoker (two of 44, 4.5%), multiple myeloma (two of 44, 4.5%), and one each of asthma, COPD, cirrhosis of the liver, lung fibrosis, and SLE. The deceased patients required a significantly longer period of mechanical ventilator (Table 2) support (10.17 ± 13.66 vs. 0.76 ± 3.18 days, *p* ≤ 0.001). One of the six deceased patients had a secondary *Klebsiella pneumonia infection.*

**Table 1:** Demographic and clinical characteristics of MERS-Cov cases by final outcome (n = 44)

Characteristics	Disease outcome		***p*** value*
	Deceased (%)	Recovered (%)	
Frequency	6 (13.6)	38 (86.3)	
Age			
Mean (SD)	59.8 (14.02)	41.16 (13.36)	
Median (IQR)	64.5 (26)	41 (21)	0.008
Gender, M:F ratio	5:01	23:15	0.65
Mechanical ventilation duration			
Mean (SD)	10.17 (13.66)	0.76 (3.18)	
Median (IQR)	4.5 (2)	0 (0)	<0.001
Hospitalization days			
Mean (SD)	40.33 (83.14)	4.76 (4.60)	
Median (IQR)	6.5 (5)	3 (3)	0.0123

*The Fisher exact test for categorical variables and the Mann-Whitney U test for continuous variables were used for association/comparison between the groups

**Table 2:** Distribution of predictors in 44 MERS-Cov patients by final outcome

Characteristics		Disease outcome		*p-*value*
	Total lesions	Deceased	Recovered	
	n (%)	n (%)	n (%)	
Frequency	44 (100)	6 (13.64)	38 (86.36)	
Chest radiograph				
Normal	30 (68.1)	0 (00.00)	30 (78.95)	<0.001
Abnormal	14 (31.8)	6(100.00)	8 (21.05)	
Disease progression				
Normal		0 (00.00)	30 (78.95)	
T1		0 (00.00)	7 (18.42)	<0.001
T2		0 (00.00)	1 (02.63)	
T3		1 (16.67)	0 (00.00)	
T4		5 (83.33)	0 (00.00)	<0.001
Lung score				
At initial presentation				
Mean (SD)	0.47 (0.99)	1.82 (1.34)	0.25 (0.75)	
Median (IQR)	0 (0.6)	1.48 (2.13)	0 (0)	<0.001
Peak of the disease				
Mean (SD)	1.39 (3.78)	8.90 (6.31)	0.20 (0.79)	
Median (IQR)	0 (0)	5.09 (9.60)	0 (0)	<0.001
Pleural effusion				
None	36 (81.82)	0 (00.00)	36 (94.74)	<0.001
Unilateral	3 (6.82)	3 (50.00)	0 (00.00)	
Bilateral	5 (11.36)	3 (50.00)	2 (05.26)	
Mechanical ventilation day				
Mean (SD)		10.17 (13.66)	0.76 (3.18)	
Median (IQR)		4.5 (2)	0 (0)	<0.001

**Fig. 1:**
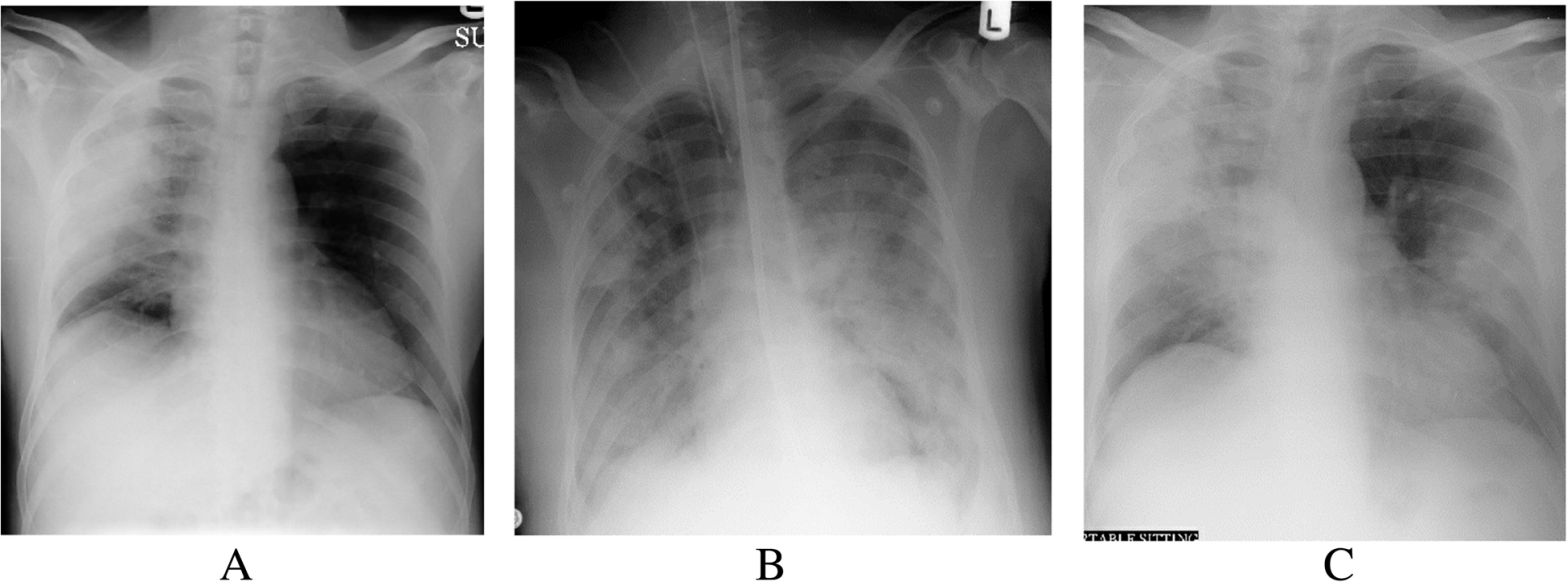
A 65-year-old man with Middle East respiratory syndrome coronavirus. Serial chest radiographs show a type 4 pattern of progression. **A** A frontal radiograph shows an ill-defined ground-glass opacity occupying the right upper and mid-zone in the peripheral location. **B** A follow-up radiograph after 3 days shows the bilateral ground-glass type of opacities with a further increase of the right opacity. **C** A follow-up radiograph after 5 days shows a further extension of the bilateral opacities with the development of consolidation in the left side along with effusion. The patient died on day 8 in the ICU

**Fig. 2:**
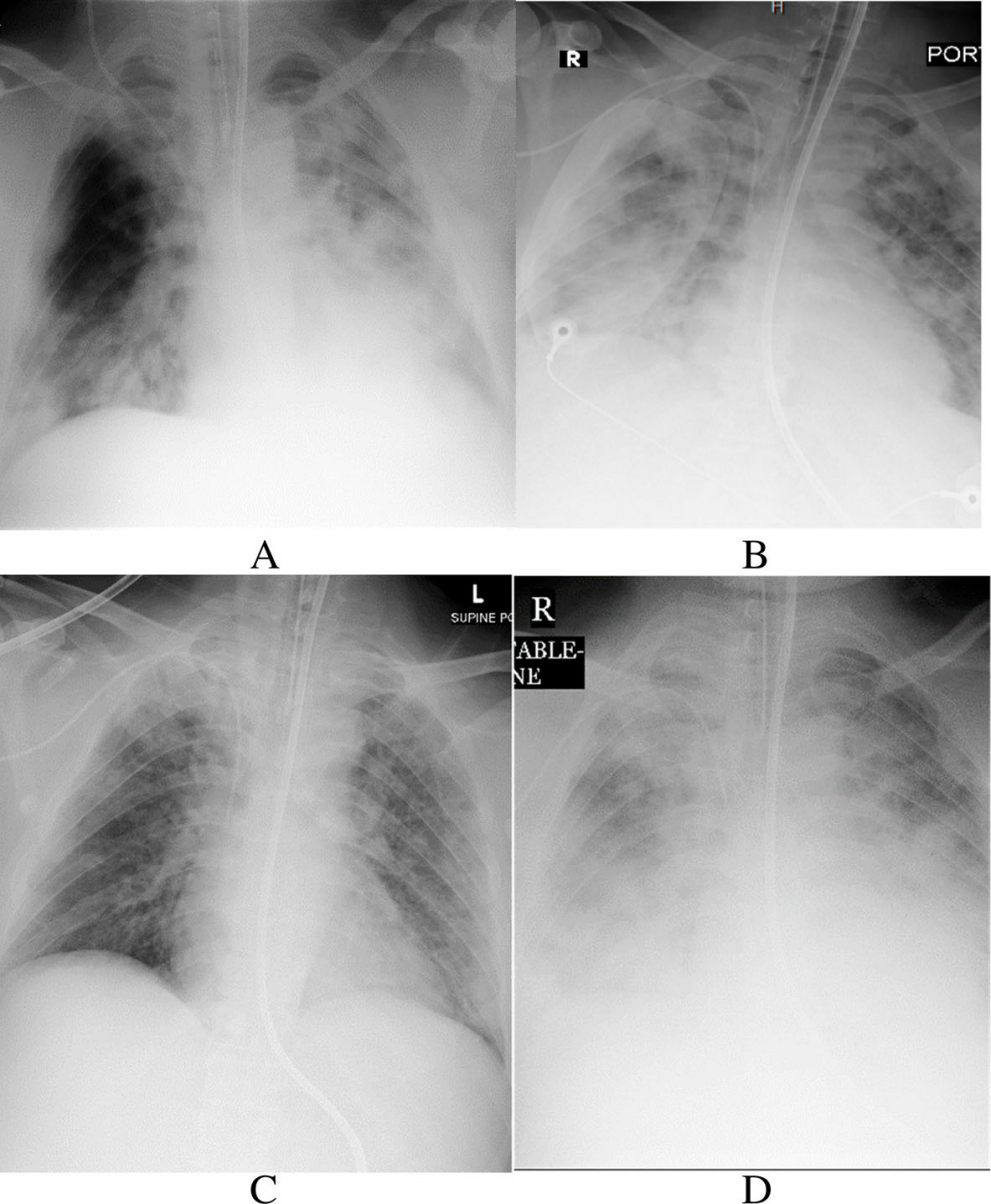
A 68-year-old man with Middle East respiratory syndrome coronavirus. Serial chest radiographs show a type 3 pattern of progression. **A** A frontal chest radiograph shows ground-glass opacity in the right lower zone with consolidation areas in the left mid and lower zone with GGO in the left upper zone. **B** On day 6, a follow-up frontal chest radiograph shows a marginal improvement of the left lung opacity with an increase of GGO in the right side. **C** A follow-up frontal chest radiograph on day 9 shows a considerable improvement of both lung opacities. **D** A follow-up frontal chest radiograph on day 12 shows a considerable improvement of both lung opacities. The patient died on the 13th day

The 44 patients underwent 182 chest radiographs during hospitalization; these were abnormal in 14 patients (31.8%) and normal in 30 patients (68.1%). Lung opacification was primarily located in the right mid and lower zone (nine of 14, 64.2%), followed by the left lower zone (three of 14, 21.4%) and left mid-zone (two of 14, 14.2%). The characteristics of the radiology features (Table 3) were GGO in the peripheral location (seven of 14, 50%), GGO and consolidation (seven of 14, 50%) (Figs. 1 and 2), pleural effusion (eight of 14, 57.1%), and air bronchogram (three of 14, 21.4%). There was an almost perfect interobserver agreement between two reviewers for detecting abnormalities on chest radiographs (*K* = 0.89, *p =* 0.001).

**Table 3:** Chest radiographic findings and disease progression of MERS-Cov cases by final outcome

Characteristics	Total lesions	Final outcome status		
		Disease outcome		*p-*value*
		Deceased	Recovered	
	n (%)	n (%)	n (%)	
Frequency	44 (100)	6 (13.64)	38 (86.36)	
Type of lung opacity				
Ground-glass opacity	9 (20.45)	6 (100)	3 (7.89)	<0.001
Consolidation	9 (20.45)	6 (100)	3 (7.89)	<0.001
Patchy consolidation	3 (6.82)	2 (33.33)	1 (2.63)	0.045
Confluent consolidation	5 (11.36)	4 (66.67)	1 (2.63)	0.001
Nodular	1 (2.27)	0	1 (2.63)	>0.999
Reticular	1 (2.27)	0	1 (2.63)	>0.999
Opacity central	1 (2.27)	1 (16.67)	0	0.136
Opacity peripheral	10 (22.73)	5 (83.33)	5 (13.16)	0.001
Pleural effusion	8 (18.2%)	6 (100)	2 (05.26)	<0.001

**Fig. 3:**
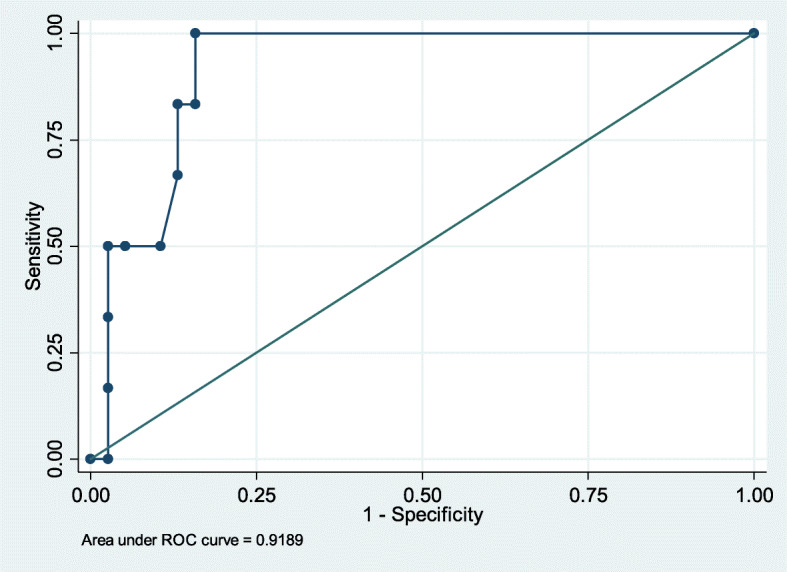
The chest radiographic score at presentation is a strong independent associated factor for mortality. The area under the curve is obtained as 0.92, and cut point 0.48 of the chest radiographic score at presentation discriminates mortality with sensitivity 100% and specificity 84.21%

The chest radiographic score at presentation was seen to be an independent and strong predictor of mortality (OR [95% confidence interval] 3.20 [1.35, 7.61]). The AUC was 0.92 at the chest radiographic score cutoff point of 0.48 at presentation and was seen to discriminate mortality with a sensitivity and specificity of 100% and 84.21%, respectively (Fig. 3). Furthermore, the deceased group had a chest radiographic score of 8.90 ± 6.31, showing the need for intensive treatment as well as close monitoring and follow-up examination. The T4 and T3 disease progression was noted in five of the 6 (83.33%) and one of the 6 (16.67%) patients with fatal outcome (Table 2).

Patients in the deceased group (6 of 44, 13.6%) were associated with significantly higher incidence of (*p* ≤ 0.02), mechanical ventilation days (*p* < 0.001), pleural effusion (*p* < 0.001), chest radiographic score (8.90 ± 6.31, *p* < 0.001), and type 3 and 4 radiographic progression of disease (*p* < 0.001) (Table 2).

## Discussion

MERS-Cov is still circulating and causing human disease in the Middle East, with isolated cases, population clusters, and nosocomial outbreaks, and there is a high risk of global spread. The current retrospective study included 44 confirmed MERS-Cov patients with a 13.6% mortality rate to establish a correlation between the mortality rate and the severity of MERS-Cov disease. The deceased group was associated with significantly higher incidence of mechanical ventilation (*p* < 0.001), pleural effusion (*p* < 0.001), chest radiographic score (8.90 ± 6.31, *p* < 0.001), and type 4 radiographic progression of disease (*p* < 0.001). The findings of the present cohort also indicated that for MERS-Cov patients, a chest radiographic score cutoff point of 0.48 at presentation suggests patient mortality, with a sensitivity and specificity of 100% and 84.21%, respectively. Additionally, a chest radiographic score of 8.90 ± 6.31 during the course of the disease necessitated aggressive therapy, as well as close surveillance and follow-up evaluation.

Because the chest radiograph is widely available, the ability to integrate chest radiographic results with clinical data collected routinely during ED admission is beneficial for ensuring rapid stratification of pulmonary parenchymal involvement. The chest radiographic score observed in the present cohort has a close relationship with the key clinical criteria used to identify patients who require hospitalization. The chest radiographic scoring system is simple to use, repeatable, and indicative of the severity of lung parenchyma involvement. It is critical to identify the most cost-effective procedures to include in ED workflow while minimizing interaction between healthcare staff and patients as well as between patients themselves.

The majority of MERS-Cov imaging research has concentrated on CT [5, 10, 11]. Even though chest radiograph is typically the first examination for patients entering the ED with suspected MERS-Cov infection, it is also characterized by simpler logistics and use; only a small number of studies have looked into its function [4, 12, 13]. The chest radiographic score is a critical parameter associated with imaging findings that have been found to be beneficial in assessing the severity of SARS, MERS-Cov, and SARS-CoV-2 infection [13]. Increased chest radiographic scores may be used as fatal prognostic markers in patients with advanced age and comorbid lung disease infected with SARS-CoV [14]. The results in the present cohort were consistent with previous research that found a higher prevalence of chest radiographic scores, pleural effusion, and pneumothorax to be linked to a poor prognosis and short-term mortality in MERS-Cov infection [15]. Although the imaging characteristics that aid in determining the prognosis of SARS-CoV-2 infection are unknown, advanced age and progressive consolidation on imaging may indicate a poor prognosis [13]. The global mortality of MERS-Cov ranges between 32 and 67% [2, 4, 16]. The mortality rate of the current MERS-Cov cohort was 13.6%. The regional variation of death rate from previously conducted studies may be skewed due to the severity of disease and smaller sample sizes than have been investigated previously. Ahmadzadeh et al. [16] affirm that the mortality example of the MERS-Cov in Saudi Arabia is not the same as that noticed in other nations in the Middle East. The differences in the virus and the genetic background of the population affected can play a role [16]. Different reasons can include a difference in the availability or ability to implement patient isolation procedures and differences in overall medical technology among involved countries [16].

The study has some inherent limitations. First, during the disease outbreak, it was most prevalent in Dubai, Abu Dhabi, and Sharjah. The number of cases seen in the remote province of the United Arab Emirates (present cohort) was limited. Second, the relatively small cohort size limited statistical analysis, which may not reveal the actual projection. Third, the visual estimation of the lung involvement was performed in one view, and this may not reflect the actual volume of the pathology in the involved lung. Finally, autopsies were not performed on any of the deceased patients.

## Conclusion

In conclusion, GGO and consolidation with a peripheral predominance on imaging are characteristic of MERS-Cov infection, and these lesions eventually spread to involve both lungs as the disease progresses. The extent of parenchymal abnormalities as determined by the radiographic lung score, the degree of radiographic progression, the presence of pleural effusion, and the number of days spent on mechanical ventilation all contribute to the clinical outcome or prognosis of coronavirus infection. The greater the extents of the chest radiographic score associated with a higher degree of disease progression (type 4), particularly in elderly or comorbid patients, may indicate a poorer prognosis in MERS-Cov infection, necessitating intensive care unit management or precluding death.

## Data Availability

Yes

## Abbreviations

MERS-Cov: Middle East respiratory syndrome coronavirus
GGO: Ground-glass opacity
SARS-CoV-2: Severe acute respiratory syndrome coronavirus 2
SARS-CoV: Severe acute respiratory syndrome coronavirus
CT: Computed tomography
ED: Emergency department
COPD: Chronic obstructive pulmonary disease
SLE: Systemic lupus erythematosus
RT-PCR: Reverse transcription polymerase chain reaction
UAE: United Arab Emirates
ARDS: Adult respiratory distress syndrome

## References

[1] Yusof MF, Eltahir YM, Serhan WS, Hashem FM, Elsayed EA, Marzoug BA, Abdelazim AS, Bensalah OKA, al Muhairi SS (2015). Prevalence of Middle East respiratory syndrome coronavirus (MERS-CoV) in dromedary camels in Abu Dhabi emirate, United Arab Emirates. Virus Genes.

[2] Hunter JC, Nguyen D, Aden B, al Bandar Z, al Dhaheri W, Abu Elkheir K, Khudair A, al Mulla M, el Saleh F, Imambaccus H, al Kaabi N, Sheikh FA, Sasse J, Turner A, Abdel Wareth L, Weber S, al Ameri A, Abu Amer W, Alami NN, Bunga S, Haynes LM, Hall AJ, Kallen AJ, Kuhar D, Pham H, Pringle K, Tong S, Whitaker BL, Gerber SI, al Hosani FI (2016). Transmission of Middle East respiratory syndrome coronavirus infections in healthcare settings, Abu Dhabi. Emerg Infect Dis.

[3] WHO (2020) Middle East respiratory syndrome coronavirus (MERS-CoV) – Saudi Arabia. https://www.whoint/csr/don/02-jul-2020-mers-saudi-arabia/en/ July.

[4] Das KM, Lee EY, Al Jawder SE (2015). Acute Middle East respiratory syndrome coronavirus: temporal lung changes observed on the chest radiographs of 55 patients. AJR Am J Roentgenol.

[5] Das KM, Lee EY, Enani MA, AlJawder SE, Singh R, Bashir S, al-Nakshbandi N, AlDossari K, Larsson SG (2015). CT correlation with outcomes in 15 patients with acute Middle East respiratory syndrome coronavirus. Am J Roentgenol.

[6] Davidson TA, Caldwell ES, Curtis JR, Hudson LD, Steinberg KP (1999). Reduced quality of life in survivors of acute respiratory distress syndrome compared with critically ill control patients. Jama.

[7] Group WM-CR (2013) State of knowledge and data gaps of Middle East respiratory syndrome coronavirus (MERS-CoV) in humans. PLoS Curr. PLoS Curr 12;5. 10.1371/currents.outbreaks.0bf719e352e7478f8ad85fa30127ddb8. 10.1371/currents.outbreaks.0bf719e352e7478f8ad85fa30127ddb8 PMC3828229 24270606

[8] Hansell DM, Bankier AA, MacMahon H, McLoud TC, Müller NL, Remy J (2008). Fleischner society: glossary of terms for thoracic imaging. Radiology.

[9] Wong KT, Antonio GE, Hui DSC, Lee N, Yuen EHY, Wu A, Leung CB, Rainer TH, Cameron P, Chung SSC, Sung JJY, Ahuja AT (2003). Severe acute respiratory syndrome: radiographic appearances and pattern of progression in 138 patients. Radiology.

[10] Hamimi A (2016). MERS-CoV: Middle East respiratory syndrome corona virus: can radiology be of help? Initial single center experience. Egypt J Radiol Nucl Med.

[11] Ajlan AM, Ahyad RA, Jamjoom LG, Alharthy A, Madani TA (2014). Middle East respiratory syndrome coronavirus (MERS-CoV) infection: chest CT findings. Am J Roentgenol.

[12] AlGhamdi M, Mushtaq F, Awn N, Shalhoub S (2015). MERS CoV infection in two renal transplant recipients: case report. Am J Transplant.

[13] Franquet T, Jeong YJ, Lam HYS, Wong HYF, Chang YC, Chung MJ, Lee KS (2020). Imaging findings in coronavirus infections: SARS-CoV, MERS-CoV, and SARS-CoV-2. Br J Radiol.

[14] Ko S-F, Lee T-Y, Huang C-C, Cheng Y-F, Ng SH, Kuo YL, Lin MC, Liu JW, Yang KD, Chen MC, Chen CL (2004). Severe acute respiratory syndrome: prognostic implications of chest radiographic findings in 52 patients. Radiology.

[15] Cha MJ, Chung MJ, Kim K, Lee KS, Kim TJ, Kim TS (2018). Clinical implication of radiographic scores in acute Middle East respiratory syndrome coronavirus pneumonia: report from a single tertiary-referral center of South Korea. Eur J Radiol.

[16] Ahmadzadeh J, Mobaraki K, Mousavi SJ, Aghazadeh-Attari J, Mirza-Aghazadeh-Attari M, Mohebbi I (2020). The risk factors associated with MERS-CoV patient fatality: a global survey. Diagn Microbiol Infect Dis.

